# The Principles and Practice of Medicine

**Published:** 1902-01

**Authors:** 


THE PRINCIPLES AND PRACTICE OF MEDICINE. Designed for the Use of Practitioners and Students of Medicine. By William Osler, M. D., Fellow of the Royal Society; Fellow of the Royal College of Physicians, London; Professor of Medicine in the Johns-Hopkins University, and Physician-in-chief to the Johns-Hopkins Hospital, Baltimore; formerly Professor of the Institutes of Medicine, McGill University, Montreal; and Professor of Clinical Medicine in the University of Pennsylvania, Philadelphia. Fourth edition. Pages, 1182; price, $6.50. New York: D. Appleton & Co. 1901.

Osler's Practice is regarded as one of the best works in the English language. There is hardly a school in the entire country that has not on its list of text-books the work of Osler. The present edition has been brought up to date and shows many changes. The article on typhoid fever has been largely rewritten and views not generally entertained are given. Malaria has received a careful revision and much new matter on the etiology and prophylaxis has been added. Many new thoughts concerning dysentery, yellow fever and the plague have also been added. Pneumonia and diphtheria have received a new page or two.

The immense clinics at the Johns-Hopkins Hospital furnish a great field for practical study, and the analyses coming from years of experience are invaluable in preparing a work like Osler's. It is impossible to name all of the changes made in this edition. It is one of the great books, and that is saying enough for it.

